# International cross-cultural development and field testing of the primary care practice questionnaire for the PaRIS survey (PaRIS-PCPQ)

**DOI:** 10.1186/s12875-024-02375-8

**Published:** 2024-05-17

**Authors:** Janika Bloemeke-Cammin, Oliver Groene, Marta Ballester, Frederico Guanais, Peter Groenewegen, Candan Kendir, Ian Porter, Amun Rehsi, Mieke Rijken, Peter Spreeuwenberg, Rosa Suñol, Jose Maria Valderas, Rachel Williams, Michael van den Berg

**Affiliations:** 1 Department Research & Innovation, OptiMedis AG, Burchardstraße 17, 20095 Hamburg, Germany; 2 Faculty of Management, Economics and Society, University of Witten/Herdecke, Witten, Germany; 3 Avedis Donabedian Research Institute (FAD), Universitat Autonoma de Barcelona, Barcelona, Spain; 4 Network for Research on Chronicity, Primary Care, and Health Promotion (RICAPPS), Barcelona, Spain; 5 OECD, Health Division, Directorate for Employment, Labour and Social Affairs, Paris, France; 6 Nivel, Netherlands Institute for Health Services Research, Utrecht, Netherlands; 7 Department of Public and Occupational Health, Amsterdam University Medical Centers, Amsterdam, Netherlands; 8 Health Services & Policy Research Group, University of Exeter, Exeter, UK; 9 Ipsos, London, UK; 10 Department of Health and Social Management, University of Eastern Finland, Kuopio, Finland; 11 Centre for Research in Health Systems Performance, Yon Loo Lin School of Medicine, National University of Singapore, Singapore, Singapore; 12 Department of Family Medicine, National University Health System, Singapore, Singapore

**Keywords:** Primary care, Chronic care, Patient-reported outcome measures, Patient-reported experience measures, Patient-centred care, People-centred healthcare system, PaRIS survey

## Abstract

**Background:**

The PaRIS survey, an initiative of the Organisation for Economic Co-operation and Development (OECD), aims to assess health systems performance in delivering primary care by measuring the care experiences and outcomes of people over 45 who used primary care services in the past six months. In addition, linked data from primary care practices are collected to analyse how the organisation of primary care practices and their care processes impact care experiences and outcomes. This article describes the development and validation of the primary care practice questionnaire for the PaRIS survey, the PaRIS-PCPQ.

**Method:**

The PaRIS-PCPQ was developed based on domains of primary care practice and professional characteristics included in the PaRIS conceptual framework. Questionnaire development was conducted in four phases: (1) a multi-step consensus-based development of the source questionnaire, (2) translation of the English source questionnaire into 17 languages, (3) cross-national cognitive testing with primary care professionals in participating countries, and (4) cross-national field-testing.

**Results:**

70 items were selected from 7 existing questionnaires on primary care characteristics, of which 49 were included in a first draft. Feedback from stakeholders resulted in a modified 34-item version (practice profile, care coordination, chronic care management, patient follow-up, and respondent characteristics) designed to be completed online by medical or non-medical staff working in a primary care practice. Cognitive testing led to changes in the source questionnaire as well as to country specific localisations. The resulting 32-item questionnaire was piloted in an online survey and field test. Data from 540 primary care practices from 17 countries were collected and analysed. Final revision resulted in a 34-item questionnaire.

**Conclusions:**

The cross-national development of a primary care practice questionnaire is challenging due to the differences in care delivery systems. Rigorous translation and cognitive testing as well as stakeholder engagement helped to overcome most challenges. The PaRIS-PCPQ will be used to assess how key characteristics of primary care practices relate to the care experiences and outcomes of people living with chronic conditions. As such, policymakers and care providers will be informed about the performance of primary care from the patient’s perspective.

**Supplementary Information:**

The online version contains supplementary material available at 10.1186/s12875-024-02375-8.

## Introduction

The growing number of people living with chronic conditions poses a major challenge to healthcare systems worldwide. To tackle this challenge, reforms of healthcare systems are needed to help people manage chronic conditions more effectively. Because primary care is usually the first point of contact between people and the healthcare system, it has the potential (realised in many countries) to play a key role in the management of chronic conditions [1, 2]. Hence, several countries worldwide have launched initiatives to expand the role of primary care in their healthcare systems, shifting chronic care management from specialist care towards primary care. Important features of a strong primary care system include care planning, care continuity, accessibility, coordination, and comprehensiveness as key facilitators of people-centred care. In short, the primary care core functions are often referred to as the 4Cs (first contact, comprehensiveness, coordination, and continuity) [3]. These are fundamental features, associated with improved health outcomes, patient satisfaction, quality of health services and lower costs [3–6]. There is evidence that a people-centred care approach, in which care delivery is designed around people’s needs, values and preferences, is key for high-quality care, particularly in the management of chronic conditions, and therefore constitutes a main pathway to address the challenge of a growing population of people living with chronic conditions [7]. Especially for people living with multiple chronic conditions, it is essential to identify personal goals in a co-creation processes between healthcare professionals and patients and align these with the clinical care goals. This practice is in line with the goal-oriented care concept, which focuses on empowerment, engagement, co-production and co-design to address the patients’ goals and priorities. Hence, goal-oriented care is seen as an approach to people-centred care delivery [8, 9]. 

Studies like the EUROPEP study, the QUALICOPC study, or the Commonwealth Fund International Health Policy Survey of Primary Care Physicians aimed to gain insight into the performance of primary care systems in Europe in terms of quality, equity and costs and to compare patients’ experiences or satisfaction with primary care across countries [10–12]. These studies were very useful to identify areas that require improvement in primary care. However, still little is known about whether people’s experiences with primary care services in different countries are consistent with the role expected of primary care. Furthermore, there is lack of comprehensive knowledge about the experiences and outcomes of care from the viewpoint of individuals living with chronic conditions across different countries. Additionally, the strength of the associations between primary care characteristics, patient experiences, and patient-reported outcomes remains unclear.

The Patient-Reported Indicators Surveys (PaRIS survey), an initiative of the Organisation for Economic Co-operation and Development (OECD), aims to fill this gap by assessing care experiences and outcomes from the perspective of people over 45 years, particularly those living with chronic conditions. The PaRIS survey aims to provide detailed patient-reported information about the experiences and outcomes of primary care considering the specific characteristics of chronic care. The results of this survey will help to better understand how healthcare systems, and particularly primary care, address the needs of people living with chronic conditions. Moreover, it will be possible to compare the results within and between the participating countries^1^, allowing international learning to further improve chronic care [13]. From the start, the PaRIS survey was conceptualised to be a methodologically rigorous initiative aimed at supporting healthcare system reform and improvement. Therefore, its development and implementation has been conducted based on priorities set by health policymakers and in close collaboration with participating countries and key stakeholders such as patients and primary care professionals [14]. The main instrument of the PaRIS survey is a questionnaire to assess outcomes and experiences with primary care services amongst primary care users aged 45 years or older. Within this population in OECD countries, at least one-third above the age of 16 years is expected to have one or more chronic condition(s), around 40% between the age of 45–64 and 60% above the age of 65 years [15, 16]. 

To complement and explain the patient-reported care experiences and outcomes, a primary care practice questionnaire (PaRIS-PCPQ) will be implemented to assess the characteristics of and care provided by the practice the service users have been in contact with, especially with regard to chronic care management. Questionnaire items focus on the assessment of facts (such as availability of procedures) rather than experiences of evaluations of care processes. The PaRIS-PCPQ plays an essential role in the PaRIS survey to understand and better explain the outcomes and experiences of people in the context of primary care systems and service delivery that cannot be derived from country-level data on primary care. It focuses on the organisation of care and processes at the practice level, which are relevant in caring for people with chronic conditions. In the PaRIS survey, eligible primary care practices are invited to complete the questionnaire. For the overall analysis of the survey, information from different levels including primary care service users, their primary care practices and country-level data will be linked to explain variations at different levels. This will help policymakers to identify the priority areas for action at different levels of the healthcare system and, supporting countries in healthcare system reforms.

This article aims to describe the development and validation of the PaRIS-PCPQ.

## Method

The development and implementation of the PaRIS survey is led by the OECD and supported by the PaRIS-SUR consortium, an international team with strong experience in primary care research and survey development [17]. Government officials from participating countries appointed their National Project Managers (NPM) to lead national teams, and work with the PaRIS-SUR consortium and the OECD to implement the survey in the respective countries. The governance structure of the PaRIS survey is published elsewhere [13]. 

In a strategic orientation phase in the beginning of the PaRIS survey, relevant indicator domains to be covered were identified. In January 2017, Health Ministers of OECD Member countries asked the OECD Health Committee to lead an effort to develop and analyse cross-country comparative measures of patients’ own experiences of medical care and health care outcomes. This mandate draws from the recommendations of a High-Level Reflection Group on Health Statistics, convened by the Health Committee in 2015 [18]. To move this forward, the OECD established an international expert taskforce to advise the Health Committee on the survey design and instruments. The first meeting of the Taskforce was held on 2–3 October 2017 and the Taskforce’s advice was taken into account in the progress report to the Health Committee meeting on 11–12 December 2017. At this meeting, the Health Committee recognised that this approach would address policy questions not yet answered by other data sources and recognised the advantages of a multi-stage design, such as the ability to link patient-reported indicators to characteristics of health care facilities and health systems, and to describe variation between health care organisations. The second meeting of the Taskforce on 12–13 March 2018 focused on several methodological issues, including the definition of eligible primary care providers and the desired concepts and indicators to be included. The advice of the taskforce laid the basis for the conceptual framework.

The conceptual framework was collaboratively developed to underpin the survey, supporting the design and development of both the primary care service users and the primary care practice questionnaire. The different domains within the framework relate to either the primary care service user or the primary care practice questionnaire. It is a unique feature of the PaRIS study that both provider and patient factors are integrated into one framework. The conceptual framework also supported the plan of analysis through a systematic, replicable, and inclusive process in close consultation with different experts and stakeholders (e.g., an international Patient advisory Panel and an international and multidisciplinary Technical Advisory Community) [13, 14, 19, 20]. The following domains (and subdomains) of the framework were identified: patient reported outcomes (symptoms, functioning, self-reported health status, health related quality of life); patient reported experiences of care (access, comprehensiveness, continuity, coordination, safety, people-centredness, self-management support, trust, overall perceived quality of care); service users’ health and health care capabilities; service users’ health behaviours (physical activity, diet, tobacco use, alcohol use), service users’ individual and sociodemographic characteristics; as well as domains of the primary care delivery system, and characteristics of the healthcare system, policy and context. The full development process is described elsewhere [21]. 

Based on the framework, the questionnaire for primary care service users and the PaRIS-PCPQ were developed in parallel. Both questionnaires and all survey processes (e.g., sampling, recruitment, data collection and analysis) were pilot tested in a field trial in 2022.

Being based on the PaRIS conceptual framework, the PaRIS-PCPQ was developed in four phases including (1) a multi-step consensus-based development of the source version of the questionnaire, (2) the translation of the English source version into the main languages of countries aiming to participate in the PaRIS survey, (3) cross-national cognitive testing, and (4) cross-national piloting the questionnaire and preliminary assessment of its reliability. Table 1 includes an overview of the development phases.

**Table 1:** Overview of the development phases of the PaRIS-PCPQ

Phase	Steps	Action
Strategic orientation (2017–2018)	Identification of priority areas for inclusion	• Discussion with OECD countries on key priority areas to guide policy reform towards more people-centred health systems.
	Definition of PaRIS conceptual framework	• Consultation with policy experts and stakeholders for the definition of the main domains in the PaRIS conceptual framework.
1. Development of the source questionnaire version (2019–2020)	Identifying existing questionnaires on primary care characteristics	• Searching and identifying existing questionnaires on primary care characteristics to be considered for further development of the PaRIS-PCPQ.
	Mapping items to the PaRIS conceptual framework	• Mapping of items of identified existing questionnaires to the PaRIS conceptual framework (domain ‘*delivery system*’).
	Eligibility rating and first questionnaire draft	• Eligibility rating of items according to predefined criteria and development of a ranking list. • Internal feedback rounds on pre-selected items. • Refinement of first item selection and development of 1st questionnaire draft.
	Feedback and modifications	• Consultation and feedback rounds with relevant stakeholders and experts of the governance of the PaRIS survey. • Rephrasing, re-structuring and shortening of the questionnaire resulting in a 2nd questionnaire draft.
2. Translation (2020–2021)	Translation of the 34-item version	• TRAP-D translation approach with a professional and a local translator including translation, review, adjudication, proofreading and documentation. • Translation of the English source version into the 17 main languages of the participating countries. • Localisation: Adaption of certain terms to ensure national applicability without risking cross-linguistic equivalence.
3. Cognitive testing (2021–2022)	Two rounds of cognitive testing	• Cognitive tests in 21 countries with more than 151 healthcare professionals across two rounds. • Source changes to improve clarity and understandability of items. • Localisation: Adaption of specific terms and concepts to increase applicability in the national context.
4. Field Trial (2022)	Pilot test and evaluation	• Pilot with 540 primary care providers in 17 countries • Descriptive and reliability analyses. • Final modifications based on the qualitative and quantitative evidence from the field trial. • Final review and sign off by the Working Party for PaRIS.

### Phase 1: development of the draft source questionnaire

The development of the PaRIS-PCPQ followed a thorough multi-step process, including the identification of existing surveys on primary care characteristics, the selection of items from the existing questionnaires based on the PaRIS conceptual framework, and stakeholder consultation and feedback for measurement modification.

The PaRIS conceptual framework was used to define the scope of the PaRIS-PCPQ. Within the framework, the questionnaire is based on the domain ‘*primary care delivery system*’ and the respective subdomains ‘*clinic characteristics*’ (referring to the subdimensions location and model of the facility, skill mix of the workforce, remuneration model, information and administrative systems, remote consultations) and the subdomain ‘*health care professional*’ (referring to the subdimensions demographics, designation, certification, chronic care training, informational and management continuity) [21]. 

#### Identifying existing surveys on primary care characteristics

From January 2020 until May 2020, we scanned the international literature for candidate questionnaires that had been used in surveys on primary care characteristics and were suitable for international use. The search strategy combined a rapid review, snowballing, expert consultation and searches in the PubMed and Google Scholar databases. Criteria for selecting questionnaires included: being used in an international context, being available in English language and being previously validated.

#### Mapping items of existing questionnaires to the PaRIS conceptual framework

The items of the pre-identified questionnaires were mapped onto the ‘*primary care delivery system*’ sub domains of the PaRIS framework following a systematic and deductive approach. It was conducted by two researchers experienced in primary care research and outcome measurement (JB and SW). In case of disagreement, a third researcher (OG) was consulted until each item was assigned to a subdomain and the corresponding subdimension of the conceptual framework.

#### Eligibility rating of preliminary items and first questionnaire draft

The items from the pre-selected questionnaires were further rated using five ad hoc a priori developed eligibility criteria: item feasibility, international comparability, response category, fact-based question, and alignment with the conceptual framework (see Table 2). A sum score was created ranging from 0 (lowest score i.e., poor eligibility) to 8 (highest score i.e., excellent eligibility).

**Table 2:** Eligibility and rating criteria

Eligibility criteria	Description/ Definition	Score^1^
Framework	Item aligns with the PaRIS conceptual framework	0 = no, 1 = yes
Fact-based	Item addresses factual circumstances	0 = no, 1 = yes
Response category	Response categories are closed-ended, examples incl: - Likert-scale - Matrix - Multiple choice	0 = no, 1 = yes
International comparability	Suitability/transferability of the item to different healthcare system contexts to allow for international comparison	0 = no, 1 = yes
Feasibility	Ease of completion, practicability of the item for a HCP to complete, conciseness/clarity of the item	0–4^2^

^1^used to develop the sum score ranging from 0 (lowest score i.e., poor eligibility) to 8 (highest score i.e., excellent eligibility)

^2^5-point Likert scale, ranging from 0 (very poor feasibility) to 4 (excellent feasibility)

The same researchers (JB, SW) rated each item according to these criteria independently. Agreement between both researchers was considered as good if the sum score differed ≤ 2 points and as poor if the sum score differed > 2 points. In case of disagreement (> 2 points), a third researcher (OG) was consulted to rate the eligibility of these items.

Following the agreement analysis, a mean sum score for each item was calculated based on the sum scores of the raters. Subsequently, a ranking was created, based on the mean sum scores, ranging from the best to the poorest performing items. In a next step, the best quartile of items within each subdimension was identified of which items were selected to be considered for inclusion in the PaRIS-PCPQ. These pre-selected items were discussed within the PaRIS-SUR consortium to gain initial feedback on item selection and question wording for the development of a first questionnaire draft. An important criterion for the design of the PaRIS-PCPQ was that it should be appropriate for an online survey to be completed by primary care professionals (e.g., physicians, nurses) or non-medical staff working in a primary care practice. The completion time was estimated at 10–15 min.

#### Feedback and modifications

Stakeholders in the PaRIS survey (including the OECD Secretariat, the OECD Health Committee, delegates to the Working Party for PaRIS, the PaRIS Patient advisory Panel, the PaRIS Technical Advisory Community and the NPMs of the participating countries) were invited to provide written or oral feedback in online meetings and bilateral meetings on the relevance, applicability, usefulness, and understandability of the items selected for the first questionnaire draft. In addition, international organisations such as World Organisation of Family doctors (WONCA), European Society for Quality and Safety in Family Practice (EQUIP) and European Forum for Primary Care (EFPC) were consulted for written feedback. Two primary care physicians were also consulted for independent review/pre-testing. The resulting second draft was translated and cognitively tested in the subsequent step.

### Phase 2: translation of the draft source questionnaire

**Fig. 1:**
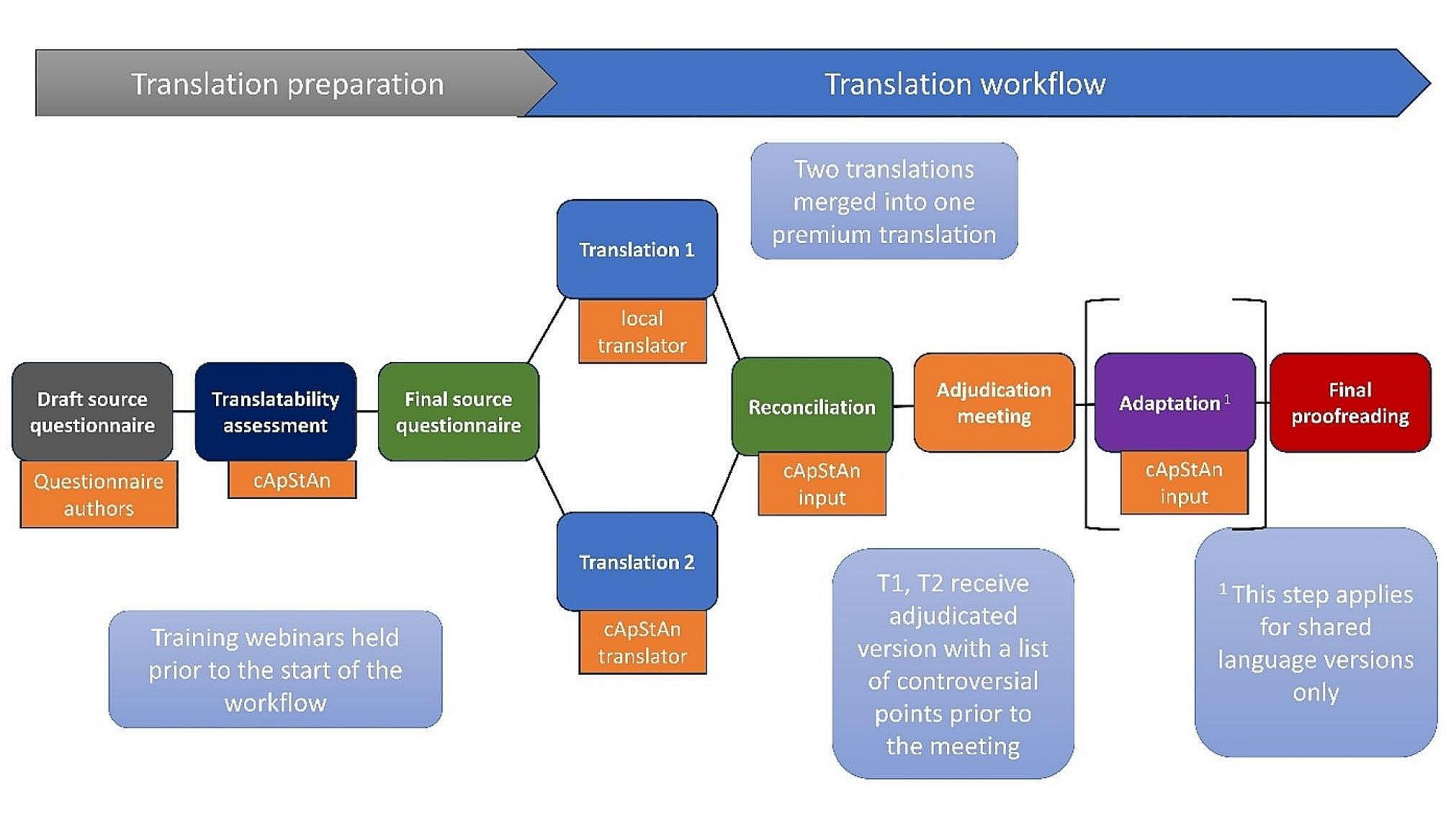
Double-translation and adjudication model following the TRAP-D approach (own illustration)

In the second phase, the source English version of the PaRIS-PCPQ was translated into the main languages of each participating country. The translation was conducted by a linguistic quality assurance company (cApStAn) in collaboration with local translators of each country using the centralised team-based approach, TRAP-D, which includes the Translation, Review, Adjudication, Proofreading and Documentation. This team-based approach to questionnaire translation and adaptation is established leading practice in international surveys [22]. The steps involved in this approach are outlined in Fig. 1.

As a first step, a translatability assessment was conducted to ensure that the source version is suitable for translation, flagging translatability problems and suggesting either alternative wording, or a translation/adaptation note to clarify a given term or expression, or to indicate to translators the type of adaptation that may be necessary. The translatability report was reviewed by the PaRIS-SUR consortium for consideration in the final source version. Subsequently, two translations were provided, one from a national translator of the respective country and one provided by cApStAn. The two translations were reconciled/merged by an adjudicator from cApStAn. Subsequently the merged translation was discussed during an adjudication meeting (one per language), which was attended by both translators, the adjudicator, the country NPM or an expert on primary care, and a member of the consortium. The aim of the meeting was to review every controversial/difficult choice to come to an agreement over the final questionnaire wording. The final questionnaire translations were then proofread by cApStAn. All stages of the translation, adaptation and reconciliation/adjudication process were documented in a monitoring tool in Excel format, the Questionnaire Translation and Adaptation Workbook (QTAW). The translation, adaption and proofread were conducted with the open source software OmegaT [23]. To support the translations a glossary was developed to increase comprehensibility of unclear terms and to facilitate comparable translations. The glossary included a description with a translation note and examples of unclear terms that were identified in the translatability assessment conducted by cApStAn as part of the translation preparation.

To ensure higher equivalence between same language versions from different countries, a list of countries having the same national language was created to identify languages with potential for collaboration (e.g., Dutch version may be used in the Netherlands and Belgium). In this case, one country produced a first line translation, and this version was adapted by another country with the same language. This shared language approach required a second ‘adaptor’ to take part in the review meeting, to implement the adaptations required for their national context and language. In addition to the source questionnaire in English, the OECD worked directly with cApStAn to produce a French reference version based on this source.

### Phase 3: cognitive testing of the draft source questionnaire

Cognitive testing was conducted to further refine the PaRIS-PCPQ and to assess eligibility, comprehensiveness, and applicability of the items within the context of the PaRIS survey, especially regarding different types of primary care providers. Furthermore, the cognitive testing was used to identify issues with translation and adaption for different national contexts to ensure that comparable data is collected across different languages and cultural and administrative contexts.

NPMs were provided with a Cognitive Testing Manual, as a practical guide, outlining the processes to follow to ensure a high quality, standardised approach to testing across all participating countries. The manual included information about sampling and recruitment, mode of interview, incentives, as well as ethical issues, such as consent, and the correct handling of data. In addition, a webinar on cognitive interviewing theory, techniques, and on the specific PaRIS survey requirements was offered by the consortium.

NPMs were asked to conduct a minimum of 10 interviews with primary care professionals in their country. Different quotas (e.g., gender, age, practice type, professional background) were recommended to ensure diversity in types of respondents. A purposive sampling method was used to recruit participants. Interviews were planned to be either conducted face-to-face or online, lasting for about an hour. A trained researcher conducted the interviews, using an interview protocol outlining the key questions to be tested and including specific probes to focus the testing on potential areas of concern (for example, reviewing terminology or probing for information about how participants understood particular items).

The testing was planned to be conducted iteratively over two rounds, allowing feedback to be considered. Researchers were encouraged to take detailed notes and to complete a structured analysis grid to ensure evidence is available to support the need for any changes throughout the two rounds of testing. In each round this led to recommendations for changes to the questionnaires. Changes that were considered were either source changes (changes or revisions to the source questionnaires to be implemented in all countries) or localisations (issues that were identified in the national context, allowing NPMs to adapt questions or response options to support comprehension, as well as to correct translation errors).

Based on the cognitive testing results, NPMs were then asked to complete a recommendations form, using the Cross-National Error Source Typology as the framework for analysis, which categorizes errors in four types: an error with the source question design, a translation error, an error in the interaction between the source question design and translation, and an issue with cultural adaptation [24, 25]. For each question tested, researchers were asked to identify the type of error, to describe the problem and to give a recommendation on how to solve the problem. After each round of testing, the Consortium systematically reviewed the recommendations and fed back to NPMs on the decisions taken and changes made.

Countries were responsible to check if ethical approval to conduct the cognitive testing was needed and if required to obtain ethical approval prior to testing.

### Phase 4: piloting of the draft questionnaires

Following the cognitive testing, the PaRIS-PCPQ was piloted as an online survey in primary care practices or facilities in which ambulatory health care services are provided to the community by health care professionals working either single-handed or within a team of health care professionals, that are licensed to serve the general population of a community, and provide ambulatory generalist medical care (i.e., in an outpatient setting), including services addressing chronic care management.

Based on these eligibility criteria, all NPMs of the participating countries were asked to construct a sampling frame and to draw a probability sample of eligible primary care practices. National representativeness was not required for the Field Trial. For all countries the NPMs were instructed to draw samples that were sufficiently large to result in a target number of 25 participating primary care practices for each country. The only exception was Iceland with a target set at 10 primary care practices given their relatively small number of PC practices.

Descriptive statistical analysis were conducted, including the analysis of the sample in terms of practice characteristics and paradata (such as completion time), as well as the distribution of responses and missing answers. Further explorative and preliminary reliability analysis were conducted to gain further insight into the performance of the PaRIS-PCPQ. For this, questions, which were expected to assess aspects of relevant constructs (latent variables), were selected a priori to construct scales. Multilevel analysis was used to construct summary scales through latent variable analysis in a three-level model with survey items at the lowest level, nested in practices, nested in countries [26]. The reliability of these scales at practice and country level was assessed, using the reliability coefficient proposed by Raudenbush for which the interpretation is equivalent to Cronbach’s alpha in a single-level model. Hence, reliability was considered acceptable when this coefficient was 0.7 or higher. The reliability in a multilevel analysis considers the agreement between respondents in their answers to survey questions relating to the same higher-level units (the calculation further includes the average number of respondents in the higher-level units and the number of items in a scale). As the PaRIS survey incorporates two levels above the individual respondents (country and primary care provider), the reliability can be calculated at both these higher levels. The reliability at country level indicates to what extent the respondents agree in their assessment of, e.g., access to primary care services in the countries of the PaRIS survey [27]. Descriptive statistics were conducted using Stata [28] and the multilevel analysis was done in MLwiN [29]. 

## Results

### Phase 1: development of the draft source questionnaire

Following the literature scan, seven a priori known primary care provider/practice questionnaires that are relevant for the PaRIS survey were selected (see Table 3).

**Table 3:** Overview of selected questionnaires

Name of the questionnaire	No. of Items	Aim of the survey
QUALICOPC [11]	60	To measure quality, costs, and equity in primary care
Assessment of Chronic Illness Care (ACIC) [30]	34	To evaluate the strengths and weaknesses of delivery of care for chronic illness in six areas and with that serving as a practical quality-improvement tool
Patient-centered Medical Home Assessment (PCMH-A) [31]	36	To evaluate the current level of a sites “medical homeness” helping to identify opportunities for improvement
Québec survey^1^ [32]	77	To measure the evolution of primary care organisations and their performance in two regions of Québec, Canada
Primary Care Assessment Tool - short version (PCAT) [33]	59	To measure the extent and quality of primary care services at a provider setting
Teamwork Assessment Survey [34]	33	To assess practice culture, relational coordination, and teamwork
Commonwealth Fund International Survey of Primary Care Physicians in 10 Nations [35]	49	To assess primary care characteristics from the perspective of primary care physicians across 10 nations

^1^ Section E (Reorganisation of primary care services) was not included, because it refers specifically to Canada with regard to the two new health reforms introduced by the Ministère de la Santé et des Services Sociaux between 2002 and 2005

Combined, the selected questionnaires provided a pool of 348 items. Following an iterative mapping process, 237 items were assigned to the subdomain of ‘*clinic characteristics*’ (location: 38 items, model: 105 items, skill mix: 11 items, remuneration: 11 items, information system: 54 items, administrative systems: 15 items, remote consultation: 3 items) and 89 items to the subdomain ‘*health care professional*’ (demographics: 8 items, designation: 1 items, certification: 0 items, chronic care training: 2 items, informational continuity: 25 items, management continuity: 32 items). 21 items referring to the workload of the health care professional or to their job satisfaction were assigned to a new subdimension named ‘general health care professional information’ which was added to the subdomain ‘*health care professional*’.

Rating scores for items showed an agreement (sum score differing by two points or less) of 80.5% (which corresponds to 280 items). Disagreement was found in 68 items (19,5%). The subsequently calculated mean sum score of each item ranged between 8 (i.e., good eligibility) to 3.5 (poor eligibility) across all domains and subdomains. The ranking list showed that items in the top quartile had sum scores of 7 or 8 (i.e., good eligibility) and were mostly from the QUALICOPC questionnaire as the source questionnaire. Details on the mapping and the eligibility rating are presented in additional material 1.

These highly ranked items of each subdimension were pre-selected and considered for further inclusion in the PaRIS-PCPQ. Following this, 70 items were pre-selected (45 items in the subdomain ‘*clinic characteristics*’ and 25 items in *’health care professional*’). After internal discussions within the PaRIS-SUR consortium on the item content and wording, questions or answering options were refined to better fit the study purpose. This resulted in a first draft questionnaire with 49 items (30 items in the subdomain ‘*clinic characteristics*’ and 19 items in the subdomain ‘*health care professional*’).

Written feedback on the first questionnaire draft was received from the OECD Secretariat, delegates to the Working Party for PaRIS, the PaRIS Patient Advisory Panel, the PaRIS Technical Advisory Community, NPMs from two countries (Canada, Norway), representatives from WONCA, EQUIP, EFPC and two primary care physicians who volunteered to review the questionnaire.

The feedback received was processed by the PaRIS-SUR consortium in consultation with the OECD Secretariat. The review and revision process took place from September 2020 to February 2021.

According to the (rather consistent) feedback received, respective questions and response categories were harmonized and refined to simplify the questionnaire and to facilitate its administration. The terminology was harmonized to improve consistency across items and to facilitate comprehensiveness and translation. Furthermore, a glossary of key terms (e.g., self-management, patient care plan) was prepared for consultation. Items that were considered too ‘physician centered’ were revised to improve their applicability to other health care professionals or non-medical employees. To reduce the length of the questionnaire, overlapping items were combined and items with marginal value removed. This resulted in a version with 34 items. As the initial structure based on the two subdomains of the conceptual framework (i.e., ‘*clinic characteristics*’ and ‘*health care professional*’) were no longer considered relevant, items addressing similar topics were combined, resulting in six (new) domains: practice profile (12 items), care coordination (11 items), chronic care management (4 items), follow-up of patients (3 items), and about yourself (4 items).

### Phase 2: translation of the draft source questionnaire

The questionnaire was translated or adapted for use in 22 countries that aimed to participate in the field trial. Translated languages were Dutch, French, Czech, Estonian, Russian, Greek, Icelandic, Hebrew, Italian, German, Norwegian, Portuguese, Romanian, Arabic, Slovenian, Welsh and Spanish. In addition, six English language versions were adapted from the original source. Details on the translated language versions and the procedure followed in each country are included in additional material 2.

Due to the nature of the PaRIS survey, which needs to reflect different healthcare systems and administrative structures, the translation process showed that the PaRIS-PCPQ required some adaptations to better fit national contexts. Hence, certain terms were localised and adapted to be more applicable to the national contexts, without losing cross-linguistic equivalence, which is a fundamental requirement in PaRIS.

The glossary and translation notes proved to be useful to facilitate translation. Terms that required adaption in many countries included the word “practice”, which has different translations in some languages, and other terms such as “case manager”, “fee for service”, “sessional fees”, “fixed honorarium”, “fixed salary”, “pay for performance”, “referral rate”, “empowerment”, “provider”, “clinical information system”, and “care plan”. During the translation process it was tried to find an equivalent that best described the term. At this stage, no structural changes (e.g., omitting a response option or whole question) were made; instead, possible structural adaptations were marked to be reviewed after the cognitive testing phase.

### Phase 3: cognitive testing of the draft source questionnaire

The questionnaire was cognitively tested in 21 countries^2^ with 151 healthcare professionals (data on the number of interviews conducted is missing from 3 countries). Most countries completed two rounds of cognitive testing; some delayed countries completed all cognitive interviews in one round. Some completed slightly fewer interviews than recommended. Most countries used a purposive sampling method, three countries applied a convenience sampling method. The interviews took place remotely, or via phone (Table 4).

**Table 4:** Overview of the cognitive testing

Country	Language	No. of interviews	No. of rounds	Mode tested	Interview type	Type of sampling
Australia	English	10	2	Online	Remote	Purposive
Belgium	French Dutch	10	2	Online	Remote	Purposive
Canada	English French	*unknown*	1	Online	Review of the online survey and complete a form	Convenience
Czech Republic	Czech	10	2	Online	Remote	Purposive
England	English	10	2	Online	Remote	Purposive
Estonia	Estonian	3	1	Online	Remote	Purposive
France	French	8	2	Online	Remote	Purposive
Greece	Greek	3	1	Phone	Remote	Convenience
Iceland	Icelandic	*unknown*	2	*unknown*	*unknown*	*unknown*
Israel	Hebrew	*unknown*	1	*unknown*	*unknown*	*unknown*
Italy	Italian	9	2	Online	Remote and in person	Purposive
Luxembourg	French German	7	2	Paper	Remote	Purposive
Netherlands	Dutch	10	2	Online	Remote	Purposive
Norway	Norwegian	10	1	Online	Remote	Purposive
Portugal	Portuguese	9	1	Online	In person	Purposive
Romania	Romanian	10	1	Online and paper	Remote	Purposive
Saudi Arabia	Arabic	10	2	Phone	Remote	Purposive
Slovenia	Slovenian	9	2	Phone	Remote and in-person	Purposive
Spain	Spanish	5	2	Online	Remote	Convenience
Switzerland	French German Italian	10	2	Paper	in-person	Purposive
Wales	English Welsh	8	2	Online Paper	Remote	Purposive

*Note* information is unknown when countries did not provide the information to the consortium

In general, the testing showed that the questionnaire was understandable and meaningful to participants. The content was relevant, and those taking part were engaged with the subject matter. However, the testing also revealed some problems in local understanding and applicability of questions mainly due to differences in healthcare systems which led to a number of changes. In that case, the Cross-National Error Source Typology recommendation form proved to be useful to document the problems, the type of error and to give a recommendation for improvement. 243 comments/recommendations were received after the first round of testing and 273 comments/recommendations after the second round of testing. Textbox 1 gives an overview of the number of comments/recommendations categorized in the different error types across all countries.

**Textbox 1:** Overview of the type of error and number of comments across all countries

**Error with the source question design** - 1st round of testing: 118 comments - 2nd round of testing: 122 comments
**Translation error** - 1st round of testing: 37 comments - 2nd round of testing: 53 comments
**Error in the interaction between the source question design and translation** - 1st round of testing: 14 comments - 2nd round of testing: 13 comments
**Issue with cultural adaptation** - 1st round of testing: 74 comments - 2nd round of testing: 85 comments

There were occasions in which question problems occurred relatively consistently across countries, which was related to a poor question design and thus required a source change in a total of 23 questions that was applied to all language versions. Relevant revisions were made to these questions to improve clarity and understandability. Box 2 gives three selected examples of the main source changes.

**Textbox 2:** Examples of source changes

Question (version that was cognitively tested)	Problem identified	Solution
‘Which of the following out-of hours options are available to your patients?’	It was not clear for respondents across the majority of countries whether the question refers to the out-of-hours options available to patients generally, or those specifically provided by their practice.	The wording of the question and response options was amended to clarify that the question refers to the out-of-hours options provided by the respective practice. If applicable an example can be added to emphasize that the question is not asking about country specific arrangements (e.g., specifying that the question is not asking on services commissioned outside the practice such as NHS 111 in the UK). Moreover, certain terms were localised to ensure that participants are answering in relation to their practice (e.g., amending ‘out of hour options’ with ‘extended working hour options’ in Portugal and England, or with ‘after-hours’ in Canada.
‘What are the roles and functions of the nurses working in your practice in chronic care management?’	In many countries (e.g., Netherlands, Switzerland) the tasks outlined in the response options of the question are also performed by other medical professionals.	Question wording was revised to make it clearer that the question refers to medical professionals in general, excluding physicians. In addition, response options were amended to better capture the tasks and functions of medical professionals in chronic care management.
‘How are individual patient care plans developed at your practice?’	The concept of a ‘care plan’ was not well understood in the majority of countries.	Based on the feedback received from the first round of testing, this question was completely revised, with two new follow-up questions developed, covering care plans in greater detail. In addition, the definition in the glossary was updated to better explain the concept of ‘care plans’. After the second round of testing, additional amendments were made to all three questions on care plans. Besides some minor tweaks of the wording, the revisions focused mainly on optimizing the response options to avoid overlaps and to better differentiate the response categories and on changing the order of response categories, so that the negative/none response option was placed at the end of the list.

In the testing, five questions were identified as less important, or not applicable and were removed for all countries. These included for example demographic questions asking for the age and sex of the respondent. Since the focus of the PaRIS-PCPQ is on the practice and not the individual professional, these seemed no longer appropriate. Source changes also included the revision of the structure and the order of the questions. After the first round of testing, two questions have been relocated to the section “chronic care management & follow-up”, as they align thematically with the content in this section. Furthermore, the question asking if the practice offers medical services to patients without an appointment was split in two questions to improve clarity.

Besides source changes that apply to all countries, country-specific localisations were made to increase applicability in the national context. For example, the question asking for the practice type was identified as problematic for most countries across both rounds of testing, with the majority citing issues of cultural transferability. In most parts this was because providers found it difficult to recognise their particular practice type in the response options provided. In addition, there was evidence of medical staff working in similar types of practices giving different answers and some reported a mix of options being available within a single primary care centre. As a result, many suggested adaptations including tweaks to the descriptions to use relevant terminology (e.g., England and Wales, ‘solo’ localised to ‘singlehanded’), and small changes to the response options were made. Other typical localisations included to localise specific terms like using the term ‘SMS’ instead of ‘text message’ in the context of remote options (for example in the Netherlands) and ‘consultations’ was localised to ‘health check’ for newly registered patients in England. Two questions referred to a ‘computerised system’ which was in some countries not well understood. In these cases, countries localised the term ‘computerised system’ to for example ‘digital system’ or ‘clinical information system’. Also, the question focusing on the professional background of the respondent was subject to localisations. Nearly all countries indicated that the professions listed in the response options were not complete or that particular professions are not appropriate for their country. Hence, NPMs were asked to specify the correct list and add additional categories that are needed.

Apart from the localisations and the source changes, seven questions were not subject to any changes.

Following the revisions based on the feedback from the cognitive testing, the questionnaire used in the field trial consisted of 32 items including 11 items in the section practice profile, 9 items in the section organisation of care (previously named care coordination), 11 items in the section chronic care management & follow-up (previously two separate sections, now combined), and one question in the section ‘about you’.

### Phase 4: piloting of the draft questionnaires

The questionnaires were scripted in an online survey format and pre-tested by the NPMs to identify any software errors. In some cases, NPMs also identified the need for further localisations to increase the applicability of the questionnaire in their country. Suggestions for localisations were taken into account; changes to the source questionnaire were not allowed anymore at this stage.

Three countries could not be included in the evaluation of the Field Trial, because they started the Field Trial later than planned and submitted the collected data later than expected to the consortium. The data presented here come from 17 countries that conducted the Field Trial in the period March to September 2022.

Most countries used a national practice registry to sample potential participating primary care practices. Where national registries of primary care practices did not exist, countries used a regional or local approach (e.g., in Canada). The NPMs contacted selected practices, either directly through e-mail, phone, or postal mail, or through an authority or regional manager to invite them to participate in the survey. Most countries used more than one mode of communication (e-mail and phone) to recruit and engage providers to participate in the Field Trial.

This recruitment resulted in 570 primary care practices from 17 countries consenting to participate in the Field Trial. Of these, 540 primary care providers completed the PaRIS-PCPQ (95.4%). Seven countries reached the target sample size (25 primary care practices). The overall response rate among primary care providers across countries was 32%, which was close to the target of 35%, though response rates varied substantially between countries [36]. The main reasons given by primary care providers for not participating was lack of time. Mainly physicians in family medicine or general internal medicine completed the questionnaire (more than 85%). Completions by other allied health professionals or practice managers were less common.

The time to complete the questionnaire was assessed in all countries except for Luxembourg, Norway, Saudi Arabia, Spain and Canada^3^, which did not provide paradata, and resulted in a median completion time of 20 min, which is slightly higher than the anticipated completion time of 10–15 min. However, the longer completion time did not lead to significant dropout and was therefore still considered acceptable. Also, outliers were identified with completion times with more than one hour in four countries, which is likely due to the daily practice of primary care (i.e., the online questionnaire is filled in in-between patient consultations or other tasks and remains open until the final questions are answered).

Descriptive statistical analysis showed that item responses were well distributed across the response options, with no indications for floor or ceiling effects. However, in five questions responses showed little variation with more than 75% of all responding primary care providers in countries having selected the same response option.

Looking in detail at these questions, this low variation was deemed reasonable from the nature of the question as it asks for common practice (e.g., the question ‘Does at least one physician or health care professional from the practice make home visits?’; response options: ‘yes’, ‘no’, ‘not sure’). Despite the limited variation in responses within countries, these questions were still considered relevant and informative because variation across countries was observed, which responds to the purpose of international learning of the PaRIS survey.

Apart from that, the proportion of missing answers in single questions was consistently below 10% across all countries. This suggests that, overall, the questions were relevant, and respondents were able to provide answers. It is important to highlight that, except for four questions, every question included a ‘not sure’ response option to mitigate potential missing responses when respondents were uncertain about their answers. Nevertheless, this option was selected by fewer than 10% of respondents in almost all questions across all countries, emphasizing the overall applicability and clarity of the questions.

There were four questions in which more than 10% of the respondents across all countries responded with ‘not sure’. Two of these questions refer to the use and functions of computerized systems, for example a clinical information system, within the practice (15% and 11% ‘not sure’). The third question asks if the practice reviews indicators for specific aspects in patient care (13% ‘not sure’) and the fourth question (11% ‘not sure’) refers to the development of patient care plans at the practice.

The increased frequency of ‘not sure’ responses could suggest either a lack of knowledge on the topic or difficulties in comprehending the question. To address the latter concern, an examination of qualitative evidence from cognitive testing was undertaken to gather additional insights for potential modifications. The qualitative data did not yield any supplementary evidence for the initial three questions, leading to the conclusion that revisions were unnecessary. However, the question related to care plans had already proven challenging during cognitive testing. Consequently, this specific question was revisited and modified to enhance overall comprehension and clarity.

For the psychometric analysis, questions that were expected to assess aspects of certain constructs were a priori grouped based on theoretical considerations and hypotheses derived from the scientific literature. This resulted in nine constructs: (1) access to primary care services (5. items), (2) time available for patient consultations (1 item with 7 sub-questions), (3) medical recordkeeping (5 items), (4) information system functionalities (3 items), (5) skill mix for chronic care management (1 item), (6) care coordination (5 items), (7) use of individual care plans (3 items), (8) Self-management support (3 items), and (9) indicators for monitoring (1 item).

Internal consistency reliability was satisfactory for all nine constructs at country level (≥ 0.72), but lower at practice level (from 0.58 for the construct ‘Access to primary care services’ to .83 for the ‘care coordination’ construct) (see Table 5). However, with a larger number of primary care practices per country participating in the PaRIS survey, the practice level reliability will automatically increase. Also, since the focus of the PaRIS survey is the comparison between countries and not between practices within countries, reliability at practice level is less important than reliability at country level to answer the main research questions of PaRIS [13]. 

**Table 5:** Reliability of the practice constructs at practice level and country level

Scales*	Reliability coefficient at practice level	Reliability coefficient at country level
Access to primary care services	0.58	0.83
Time available for patient consultations	0.73	0.92
Medical recordkeeping	0.79	0.91
Information system functionalities	0.78	0.95
Care coordination	0.83	0.87
Use of individual care plans	0.63	0.72
Self-management support	0.65	0.81

^*^The constructs ‘skill mix’ and ‘indicators for monitoring’ are individual items and therefore not included here

Taken the quantitative and qualitative evidence together, the questionnaire was subject to final modifications. This included minor modifications in the wording of four questions to improve clarity and understanding, amending the response options of two questions, and adding two new questions on care plans to better capture the concept of care plan development and use. This resulted in the final source version with 34 items. The questionnaire is available on the OECD PaRIS Website (https://www.oecd.org/health/paris/)^4^.

## Discussion

This article describes the development of the PaRIS-PCPQ for the PaRIS survey including its pilot testing in an international Field Trial. The resulting questionnaire aims to profile and characterise primary care practices across participating countries and the delivery of primary care services with a special focus on chronic care management in primary care. In combination with the PaRIS survey on patient experiences and outcomes, it can shed light on the performance of countries’ primary care services in terms of chronic care delivery, and on how patient-reported care experiences and outcomes relate to certain practice characteristics [37]. 

Because of the scarcity of available instruments that reliably measure primary care practice characteristics for healthcare system performance, the PaRIS-PCPQ is of significant value. The thorough development process described, in particular the translation and localisation process, can serve as a best-practice example on how to conduct these important steps in survey development on an international level. The PaRIS-PCPQ can also be used in other countries that aim to participate in future waves of the PaRIS survey, with the possibility of a comparison to other countries.

Developing a questionnaire for an international survey on primary care poses multiple challenges. A one-size-fits-all approach is at risk of insufficiently covering the complexity of primary care with its diverse organisational, information and communications technology and purchasing arrangements [38]. The use of different terminologies and concepts (e.g., the term “primary care” alone varies largely across countries) poses additional challenges and required detailed translation notes on the meaning and scope of the questions.

To overcome these challenges, we went through rigorous development phases with a multi-step approach and involved key stakeholders on multiple occasions [14]. Inclusive development of the PaRIS survey together with patients and providers is a key principle of the survey. Also, the development of the PaRIS-PCPQ has been a collaborative approach to ensure comprehensiveness and relevance to all main stakeholders.

To make sure the questionnaire is suitable to the national and cultural context of participating countries and that included terms and concepts are well understood, the translation was an important step in the development of the PaRIS survey. In the translation process of the PaRIS-PCPQ, it became apparent that many more localisations were necessary than expected because terms, descriptions and structures varied substantially across countries. Initially, we aimed to limit localisations as much as possible to maintain comparability across countries. However, if a certain translation is comparable linguistically, but does not fit in the country context, it will still lead to unreliable data that is not comparable across countries. Hence, localisations (which may be viewed as local deviations) had to be applied to maintain comparability. By using a collaborative and iterative approach as foreseen in the TRAP-D approach, high-quality translations were provided that accurately reflect the local context while maintaining the integrity of the original source. It allowed to translate certain terminologies and concepts (e.g., patient care plans) correctly and meaningfully. Through the centralised approach it also provided greater consistency between national versions [22, 39]. A final proof check of each version at the end of the translation process ensured that any minor residual typographical errors, punctuation or spelling issues in the translation were corrected.

In addition to the translation process, cognitive testing is key in international studies to ensure comparable data is collected across different languages, cultural and administrative contexts. In the PaRIS survey, cross-national cognitive testing was applied to identify any problems with the source questionnaire, translated and adapted items [40]. Since the PaRIS-PCPQ is based on existing measures that have been tested and validated (see Table 3), the focus of the testing was on how particular questions work within the context of the PaRIS survey. However, although several items were taken from existing and validated questionnaires, some terms (e.g., clinical information system, care plans) were not necessarily well known in the participating countries. It might be because the existing questionnaires on which the PaRIS-PCPQ is based were mostly used in English speaking countries (except the Commonwealth Fund Survey and the QUALICOPC survey), or because the questions were not suitable for the respondents. The PaRIS-PCPQ is designed to be completed by a range of primary care professionals, including physicians, nurses or other health professionals, and non-medical staff such as practice managers. As such, the questions need to be applicable and understandable to all these persons with a different professional background. Hence, the cognitive testing revealed that many modifications were needed to come to a questionnaire that, on the one hand, respects the national contexts and different respondents, and on the other, is suitable for use in an international survey. Conducting two rounds of cognitive testing was therefore a sensible approach to allow iterative feedback and to test modifications of the first round again.

Initially it was planned to conduct the translation and cognitive testing simultaneously in countries. However, due to general delays in the project and working with countries as well as to the impact of the COVID-19 pandemic, countries followed different timelines, which complicated the management process. Due to the delays, it was also not possible to test the English source version first, to identify issues with the source that could have been addressed prior to translation and cognitive testing. This would have probably reduced the number of issues identified with the source questionnaires and focus the testing more on translation and cultural adaptation.

Field-testing is another key step in questionnaire development. A total of 540 practices participated in a Field Trial and completed the PaRIS-PCPQ. The quantitative data were useful to assess preliminary psychometric properties of the questionnaire, resulting in final modifications of the questionnaire and a final version to be used in the PaRIS survey.

Questionnaires that assess practice characteristics in the field of primary care and that are suited for international comparisons are rare. A well-known questionnaire is the one developed in the international QUALICOPC survey, which aimed to assess the quality, costs and equity in primary care in 34 countries [11]. Similar to the PaRIS-PCPQ, the general practitioner (GP) questionnaire of the QUALICOPC survey is based on existing questionnaires. However, the cognitive testing and pilot test were conducted in one step in the QUALICOPC study and in only three countries, which the authors declare as a limitation in the development process [41]. Besides, QUALICOPC started in 2010 and the structure and organization of primary care evolved considerably since then.

Other studies that report on survey results of primary care physicians, such as the Commonwealth Fund International Health Policy Survey of Primary Care Physicians in 2009 and 2019, are lacking a detailed description of the development of the questionnaire [42–44]. Furthermore, most of these questionnaires were designed to be completed by individual primary care physicians and, unlike PaRIS, do not focus on the practice level, with the possibility to be completed by other staff of the PC practices. In addition, the PaRIS-PCPQ focuses on factual descriptions of structures and processes in primary care rather than views, attitudes or perceptions of professionals working in primary care [45, 46]. Although such surveys are very useful, they do not correspond to the purpose and research questions of PaRIS.

In summary, the rigorous development of the PaRIS-PCPQ, including multi-step consensus-based exercise, a collaborative and iterative translation approach, extensive cross-national cognitive testing in two rounds, cross-national field-testing, and involvement of stakeholders throughout the development process with many quality checks make the development of the PaRIS-PCPQ unique. This process resulted in a robust questionnaire for use in the PaRIS survey and other international comparative studies on primary care organisation and performance.

## Conclusions

The PaRIS-PCPQ plays an essential role in the PaRIS initiative to understand how the outcomes and care experiences of people with chronic conditions relate to key characteristics of primary care practices. Practice-level information generated with this questionnaire will help to interpret variations in patient-reported outcomes and care experiences and in the identification of good practice. Engaging stakeholders early in the process and involving them in the design and development of the survey, the rigorous translation process, cross-national cognitive testing and large-scale piloting helped to ensure international comparability while respecting national contexts. Results from this questionnaire will provide guidance for policy and practice development in countries, to improve the quality of primary care for people living with chronic conditions.

### Electronic supplementary material

Below is the link to the electronic supplementary material.

Supplementary Material 1

Supplementary Material 2

## Data Availability

The data from the Field Trial cannot be shared by the authors, as they are owned by the participating countries. Furthermore, these data were collected exclusively for developing the PaRIS survey and its survey instruments; they cannot be used for substantive analysis as the data collection did not aim at providing valid and representative data for the countries and providers involved.

## Footnotes

1 Australia, Belgium, Canada, Czech Republic, France, Greece, Iceland, Israel, Italy, Luxembourg, Netherlands, Norway, Portugal, Romania, Saudi Arabia, Slovenia, Spain, Switzerland, USA, Wales.

2 The United States implemented PaRIS in a national ongoing initiative (Medicare Current Beneficiary Survey) concerning patient outcomes and experiences and did not plan to collect data from primary care practices and therefore did not participate in cognitive testing.

3 Canada did not complete the data collection at the time of the analysis and was only included in the description of the sample.

4 https://www.oecd.org/health/paris/PaRIS-provider-questionnaire.pdf.

## Authors information

The views expressed and arguments employed herein are solely those of the author(s) and do not necessarily reflect the views of the OECD or its Member countries. The Organisation cannot be held responsible for possible violations of copyright resulting from the posting of any written material on this website.

## Abbreviations

EFPC: European Forum for Primary Care
EQUIP: European Society for Quality and Safety in Family Practice
NPM: National Project Managers
OECD: Organisation for Economic Co-operation and Development
PaRIS: Patient-Reported Indicator Surveys
PaRIS-PCPQ: PaRIS Primary Care Practice Questionnaire
QTAW: Questionnaire Translation and Adaptation Workbook
TRAP-D: Translation, Review, Adjudication, Proofreading and Documentation
WONCA: World Organisation of Family Doctors
