# Supplementary material for: International cross-cultural development and field testing of the primary care practice questionnaire for the PaRIS survey (PaRIS-PCPQ)
Source: BMC Prim Care. 2024 May 17;25:168. doi: 10.1186/s12875-024-02375-8 (PMC11100105; doi:10.1186/s12875-024-02375-8)
Supplement: Supplementary file 2 — Supplementary Material 2 [file 12875_2024_2375_MOESM2_ESM.docx]

**Additional Material 2**

Overview of PaRIS translations and procedure followed

|  | **Country** | **Language** | **Procedure** |
| --- | --- | --- | --- |
| 1 | Australia | English | Adaptation from source by country |
| 2 | Belgium | French | Adaptation from OECD reference version |
|  | Belgium | Dutch | Adaptation from Dutch (Netherlands) |
| 3 | Canada | English | Adaptation from source by country |
|  | Canada | French | Adaptation from OECD reference version |
| 4 | Czech Republic | Czech | Translation |
| 5 | England | English | Adaptation from source by country |
| 6 | Estonia | Estonian | Translation |
|  | Estonia | Russian | Translation |
| 7 | France | French | Adaptation from OECD reference version |
| 8 | Greece | Greek | Translation |
| 9 | Iceland | Icelandic | Translation |
| 10 | Israel | Hebrew | Translation |
| 11 | Italy | Italian | Translation |
| 12 | Luxembourg | French | Adaptation from OECD reference version |
|  | Luxembourg | German | Adaptation from German (Switzerland) |
|  | Luxembourg | English^1^ | Adaptation from source by country |
|  | Luxembourg | Portuguese^1^ | Adaptation from Portuguese (Portugal) |
| 13 | Netherlands | Dutch | Translation |
| 14 | Norway | Norwegian (Bokmal) | Translation |
| 15 | Portugal | Portuguese | Translation |
| 16 | Romania | Romanian | Verification |
| 17 | Saudi Arabia | Arabic | Translation |
| 18 | Slovenia | Slovenian | Translation |
| 19 | Spain | Spanish (Castellano) | Translation |
| 20 | Switzerland | French | Adaptation from OECD reference version |
|  | Switzerland | German | Translation |
|  | Switzerland | Italian | Adaptation from Italian (Italy) |
| 21 | USA | English | Adaptation from source by country |
| 22 | Wales | Welsh | Verification |
|  | Wales | English | Adaptation from source by country |
